# The predictive value of multi-phase contrast-enhanced MRI for pathological complete response after neoadjuvant chemoradiotherapy in rectal cancer

**DOI:** 10.3389/fonc.2026.1834468

**Published:** 2026-05-20

**Authors:** Jingjing Xie, Kaihe Lin, Suying Wu, Jiajun Lin

**Affiliations:** Department of Radiology, The First Hospital of Putian City, Putian, Fujian, China

**Keywords:** diagnostic efficacy, multi-phase contrast-enhanced magnetic resonance imaging, neoadjuvant chemoradiotherapy for rectal cancer, optimal imaging phase, pathological complete response

## Abstract

**Background:**

Accurate prediction of pathological complete response (pCR) after neoadjuvant chemoradiotherapy (NCRT) for rectal cancer is crucial for determining subsequent treatment strategies. Although studies have shown that single-phase contrast-enhanced (CE) imaging can accurately assess complete response status after NCRT, the diagnostic value of different phases in multi-phase CE MRI remains unclear.

**Methods:**

A retrospective cohort study was conducted involving patients with locally advanced rectal cancer who underwent total mesorectal excision (TME) after NCRT at our institution between August 2020 and October 2025. Two radiologists, blinded to individual, clinical, and histopathological information, independently assessed enhancement characteristics on multi-phase CE MRI. The diagnostic efficacy of each imaging phase for predicting pCR was compared to identify the optimal phase. Multivariate logistic regression analysis was conducted to explore independent predictors of pCR.

**Results:**

A total of 68 patients (mean age 64.8 ± 8.5 [standard deviation]) were included. The third phase of the CE (CE-3) MRI series (approximately 76 seconds) demonstrated optimal performance in predicting pCR, with an accuracy of 0.882, specificity of 0.925, and an area under the receiver (AUC) of 0.829 (95% CI: 0.708–0.950). Inter-observer agreement was favorable (κ = 0.672, 95% CI: 0.461–0.886). Compared with T2-weighted imaging combined with diffusion-weighted imaging (T2DWI), CE-3 MRI demonstrated superior accuracy in identifying treatment response (0.882 vs 0.647, p=0.002) and a superior ability to identify patients with residual tumor (Net Reclassification Index [NRI] for ypT1–4 group =0.339, 95%CI: 0.204–0.482, p<0.001). Multivariate logistic regression analysis demonstrated that the yT stage based on CE-3 MRI was the sole independent predictor of pCR after NCRT (P < 0.001).

**Conclusion:**

CE MRI can identify residual tumor more precisely. In particular, CE-3 MRI series demonstrated superior diagnostic efficacy and good inter-observer agreement, which helps prevent missed diagnoses and provides clinicians with a more stable and reliable imaging assessment protocol.

## Introduction

Colorectal cancer is a highly prevalent malignancy worldwide, with rectal cancer accounting for approximately 38.9% of cases (1). The International Agency for Research on Cancer projects that by 2040, the global incidence of colorectal cancer will reach 3.2 million new cases, with approximately 1.6 million deaths, indicating a persistently increasing disease burden (1, 2). Although the widespread adoption of colorectal cancer screening has partially reduced overall incidence rates, a significant proportion of patients are still diagnosed at an advanced stage (3–5). Neoadjuvant chemoradiotherapy (NCRT) combined with total mesorectal excision (TME) remains the primary treatment strategy for locally advanced rectal cancer (LCRT). However, surgery is frequently associated with complications such as loss of anal function, urinary dysfunction, and sexual dysfunction, severely impacting patients’ quality of life (6). Studies indicate that approximately 15%–27% of patients achieve pathological complete response (pCR) after NCRT. Adopting a “ watch-and-wait “ approach for such patients can avoid surgery and its associated complications (7–10). The key to implementing this strategy lies in the precise identification of pCR patients.

High-spatial-resolution T2-weighted imaging (T2WI) combined with diffusion-weighted imaging (DWI), referred to as T2DWI, holds significant value for evaluating treatment efficacy in rectal cancer patients after NCRT (11–13). However, fibrosis, oedema, and inflammatory responses frequently accompany NCRT. As their magnetic resonance imaging (MRI) signals often overlap with those of microscopic tumor residues, this limits the accuracy of NCRT efficacy assessment (reported accuracy around 0.63) and introduces substantial inter-observer variability (κ = 0.247 to 0.54) (14–18). Therefore, there is an urgent need to identify more precise and stable imaging protocols to improve the detection rate of pCR and identify minute residual tumors. Recent studies indicate that CE MRI can accurately identify minute residual tumors after NCRT and improve the accuracy of diagnosing pCR (19, 20). However, these studies primarily selected single-phase MRI images from multi-phase CE MRI sequences based on clinical experience for discussion. It remains unclear whether this conclusion applies to other phases, and no systematic comparison of all dynamic phases has been reported. This study addresses this gap by comprehensively evaluating all eight phases of multi-phase CE MRI to identify the phase with the best predictive performance for pCR, thereby providing an evidence-based rationale for optimizing imaging protocols in post-NCRT assessment.

## Materials and methods

### Study patients

This single-center retrospective study was approved by the Medical Ethics Committee of our institution (approval number 2026-002), with the requirement for informed consent waived. All patients were rectal cancer patients treated at our hospital between August 2020 and October 2025. Inclusion criteria: Patients with biopsy-confirmed LARC, diagnosed on MRI as either clinical stage T3–T4 or any T stage with positive lymph node metastasis status, who underwent NCRT followed by TME. Exclusion criteria: Absence of post-NCRT CE MRI or poor MRI quality for assessment.

### MRI examination

All patients underwent MRI using a 3.0T superconducting magnetic resonance imaging system (Skyra; Siemens Healthcare). Patients were positioned head-first and supine to acquire axial T2-weighted imaging (T2WI), diffusion-weighted imaging (DWI), and contrast-enhanced (CE) MRI of the rectum. All sequences utilized a small field-of-view with high resolution, featuring a slice thickness of 3.0 mm and no inter-slice gap. Multi-phase CE MRI commenced at the 19th second following contrast agent injection via a high-pressure syringe (Gd-DTPA, 0.1 mmol/kg, 2.0 ml/s, Sichuan Guorui Pharmaceutical), capturing 8 phases, each lasting 23 seconds, with a k-space center refill time of 11 seconds (Detailed scanning parameters are provided in **Supplementary Table 1**).

### MRI feature evaluation

Baseline MRI features included: T stage, N stage, extramural venous invasion (EMVI), mesorectal fascia invasion (MRF), tumor involvement as a percentage of the intestinal circumference, and distance from the tumor base to the anal margin. Post-NCRT assessment included: yT stage, N stage, EMVI, and MRF.

Observers assessed the yT stage across all phases of the multi-phase CE MRI series following NCRT. A second assessment round, conducted at least four weeks later, determined the yT stage based on high b-value diffusion-weighted imaging (b=1500 sec/mm²) combined with T2-weighted imaging (T2DWI).

MRI features were independently evaluated by two experienced radiologists (Reader J.J.X. and Reader J.J.L., with 4 and 10 years of MRI diagnostic experience, respectively). Where discrepancies arose between the two assessors, a third senior radiologist (Reader S.Y.W., with 20 years of MRI diagnostic experience) served as an arbitrator. All readers were blinded to patient demographics, clinical, and pathological information.

On MRI, a complete response after NCRT for rectal cancer is characterised by restoration of normal intestinal wall layers, or the presence of only a thin layer of T2-hypointense fibrotic scarring in the tumor region. Conversely, the presence of tumor signal or thick fibrotic scarring with restricted diffusion suggests residual tumor (21). Miao et al. further indicated that post-contrast MLE constitutes a significant indicator of mucosal structure restoration after NCRT (19). Consequently, in the CE MRI assessment of this study, the absence of abnormal enhancement or the presence of only thin linear enhancement (≤2 mm in thickness) within the primary lesion area was interpreted as pathological complete response.

**Figure 1:**
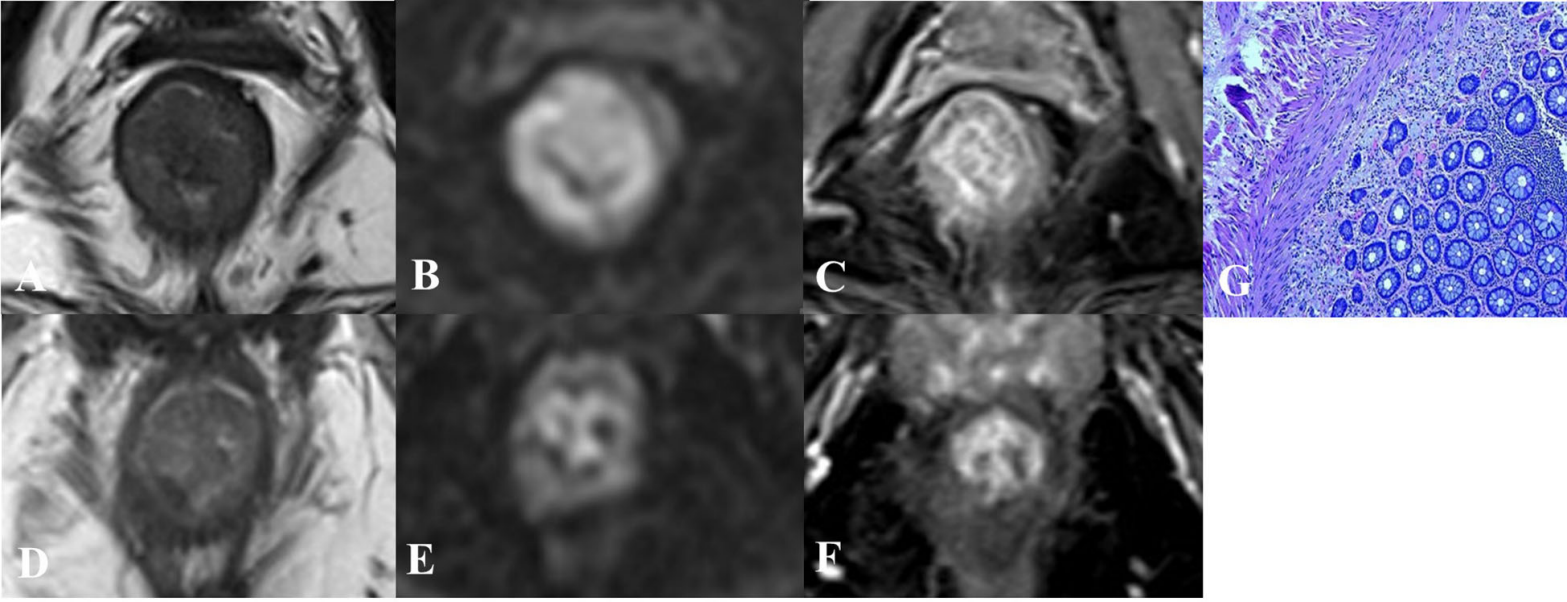
Imaging and pathological findings in a 74-year-old female with low rectal adenocarcinoma. **(A–C)** Baseline axial T2-weighted imaging, diffusion-weighted imaging (DWI), and contrast-enhanced MRI demonstrate a nearly circumferential tumor with intermediate T2 signal, restricted diffusion, and contrast enhancement. **(D–F)** After completion of neoadjuvant chemoradiotherapy, follow-up imaging shows no significant mucosal thickening in the original lesion area. DWI reveals no diffusion restriction, and contrast-enhanced MRI depicts normal mucosal enhancement. **(G)** Postoperative pathological examination of the mesorectal specimen confirmed a complete pathological response, with no residual tumor tissue identified.

**Figure 2:**
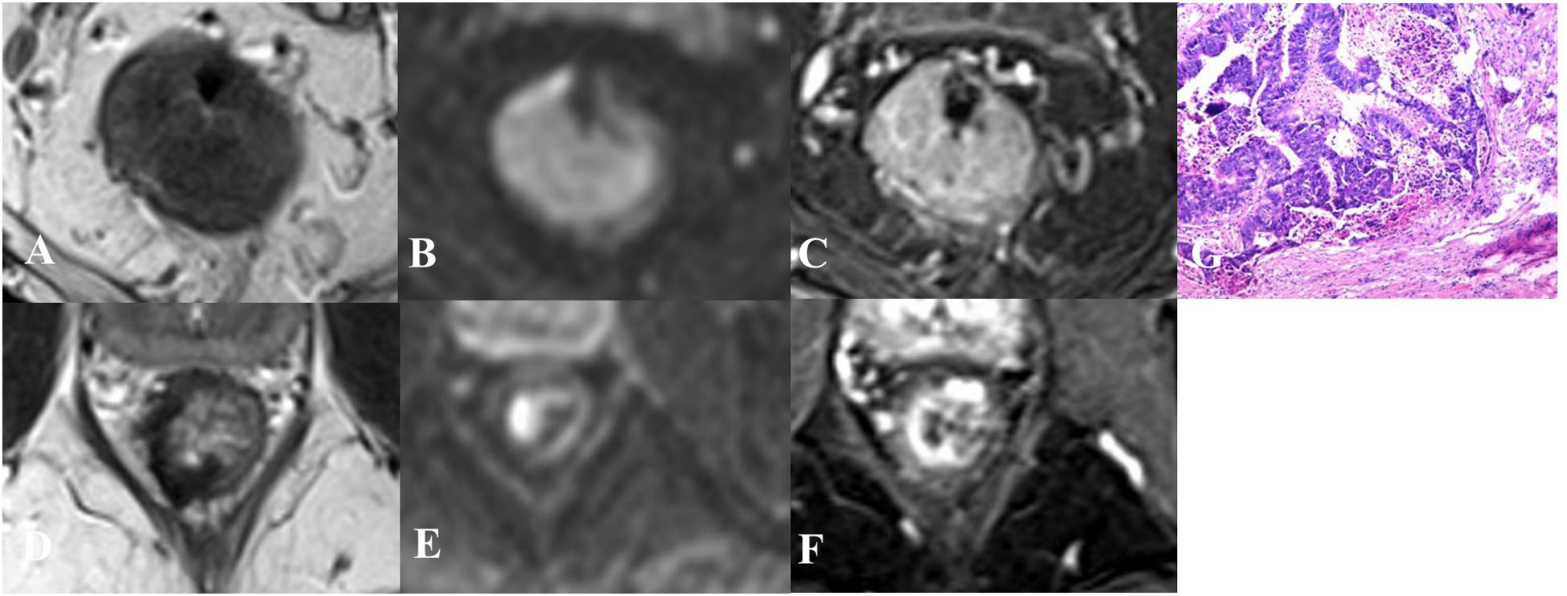
Imaging and pathological findings in a 71-year-old male with low rectal adenocarcinoma. **(A–C)** Baseline axial T2-weighted imaging, diffusion-weighted imaging (DWI), and contrast-enhanced MRI show a tumor occupying &gt;3/4 of the bowel circumference, characterized by intermediate T2 signal, restricted diffusion, and contrast enhancement. **(D–F)** After neoadjuvant chemoradiotherapy, the lesion area exhibits thickened, hypointense changes on T2-weighted imaging, persistent restricted diffusion on DWI, and rigid, thickened enhancement on contrast- enhanced imaging. **(G)** Postoperative pathological examination following total mesorectal excision confirmed the presence of residual tumor tissue within the submucosa and muscularis propria.

The imaging criteria for yT0 group and yT1–4 group are as follows (**Figures 1**, **2** illustrate examples):

1. CE MRI: yT0 group: No abnormal enhancement in the mucosa or thin-layer linear enhancement (thickness ≤ 2 mm), with no abnormal nodular enhancement beyond the mucosal layer. yT1–4 group: Focal thick-slice (thickness > 2 mm) or nodular abnormal enhancement is observed.
2. T2DWI: yT0 group: No distinct tumor signal visible on T2WI, with no focal hyperintensity on DWI in the corresponding region. yT1–4 group: Residual tumor signal is demonstrated on T2WI, and/or focal hyperintensity is present on DWI in the corresponding region; or thickened fibrosis is observed, accompanied by focal hyperintensity on DWI in the corresponding region.

### Pathologic assessment

All patients underwent TME following completion of NCRT. Postoperative pathological specimens were assessed for pathological T (ypT) staging according to the American Joint Committee on Cancer guidelines (22).

### Statistical analysis

Continuous variables were compared using the independent t-test or Mann–Whitney U test, and categorical variables using the chi-square or Fisher’s exact test.

To identify the optimal contrast-enhanced phase for predicting pCR while avoiding type I errors inherent in multiple pairwise comparisons, a two-step approach was adopted. First, a descriptive ranking method based on multiple performance metrics—including accuracy, sensitivity, specificity, positive predictive value, negative predictive value, area under the receiver operating characteristic curve (AUC), and inter-observer agreement (Cohen’s κ; κ ≤ 0.20: poor; 0.21–0.40: fair; 0.41–0.60: moderate; 0.61–0.80: good; 0.81–1.00: excellent)—was used to select the single best phase. Emphasis was placed on specificity and reproducibility to ensure safe application in a “watch-and-wait” strategy.

Second, the selected optimal phase was formally compared with each of the other phases using DeLong tests for AUC differences and Net Reclassification Improvement (NRI) analyses. Adjustments for multiple comparisons were applied accordingly to control the type I error rate.

This optimal phase was then compared with the T2DWI protocol. The DeLong test was used to compare AUCs; integrated discrimination improvement (IDI) and NRI were employed to assess stratification capacity; and McNemar’s test was applied to evaluate differences in accuracy, sensitivity, and specificity.

Univariate logistic regression was performed on all potential clinical and imaging variables. Variables with *P* < 0.05 were entered into multivariate logistic regression using forward stepwise selection. All analyses were performed using R software (version 4.5.0), with *P* < 0.05 considered statistically significant.

## Results

### Patient characteristics

**Figure 3:**
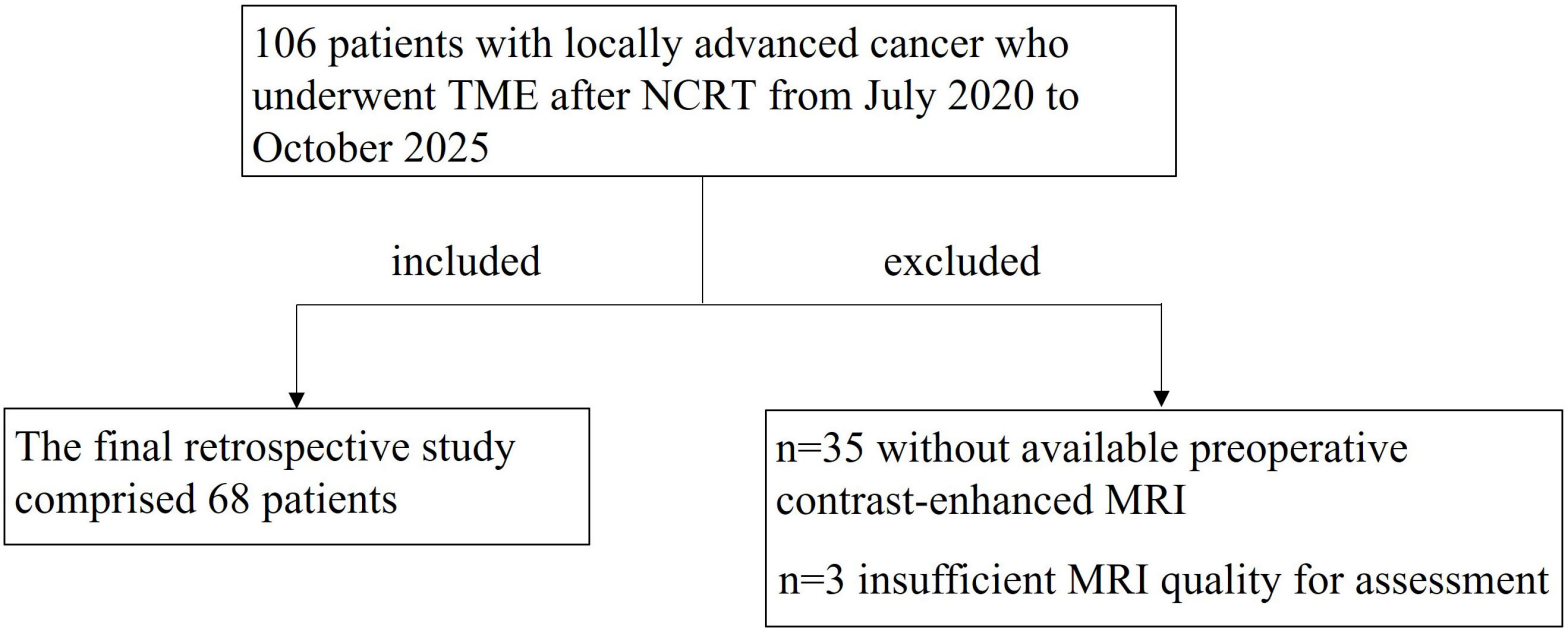
Patient inclusion and exclusion protocol. NCRT, neoadjuvant chemotherapy and radiation therapy; TME, total mesorectal excision.

This retrospective study initially enrolled 106 rectal cancer patients who underwent TME after NCRT at our institution between August 2020 and October 2025. Among these, 35 were excluded for absence of post-NCRT contrast-enhanced MRI, and 3 were excluded for insufficient MRI quality for assessment(**Figure 3**). Ultimately, 68 patients were included in the study, comprising 50 males and 18 females, with a mean age of 64.8 ± 8.5 years (mean ± standard deviation). Of these, 15 patients had confirmed ypT0 status (pCR for primary tumor). No statistically significant differences were observed between the ypT0 and ypT1–4 groups regarding age, gender, tumor location, proportion of intestinal circumference involved (TIL), or the presence of MRF, EMVI or N stage before and after NCRT (all *P* > 0.05) (**Table 1**).

**Table 1:** Patient and tumor characteristics stratified by pathological response after neoadjuvant chemoradiotherapy.

Characteristic	Total (n=68)	ypT0 group (n=15)	ypT1–4 group (n=53)	*P* value
Mean Age (y)	64.8 ± 8.5	64.0 ± 10.1	65.1 ± 8.2	0.713
Sex				0.093
Male	50 (73)	8 (53)	42 (79)	
Female	18 (27)	7 (47)	11 (21)	
TIL				0.731
<1/2	6 (9)	2 (13)	4 (8)	
1/2-3/4	18 (26)	4 (27)	14 (26)	
>3/4	44 (65)	9 (60)	35 (66)	
Tumor location				1.000
Low	21 (31)	5 (33)	16 (30)	
Middle	38 (56)	8 (53)	30 (57)	
Upper	9 (13)	2 (13)	7 (13)	
Pre-NCRT T stage				0.426
T2	9 (13)	3 (20)	6 (11)	
T3	43 (63)	10 (67)	33 (62)	
T4	16 (24)	2 (13)	14 (26)	
Pre-NCRT EMVI				0.530
Negative	49 (72)	12 (80)	37 (70)	
Positive	19 (28)	3 (20)	16 (30)	
Pre-NCRT MRF				1.000
Negative	40 (59)	9 (60)	31 (58)	
Positive	28 (41)	6 (40)	22 (42)	
Post-NCRT EMVI				0.185
Negative	60 (88)	15 (100)	45 (85)	
Positive	8 (12)	0 (0)	8 (15)	
Post-NCRT MRF				0.672
Negative	59 (87)	14 (93)	45 (85)	
Positive	9 (13)	1 (7)	8 (15)	
Pre-NCRT N stage		55		0.409
N0	39 (57)	10 (67)	29 (55)	
N1-2	29 (43)	5 (33)	24 (45)	
Post-NCRT N stage				0.055
N0	56 (82)	15 (100)	41 (77)	
N1-2	12 (18)	0 (0)	12 (23)	

Data in parentheses are percentages, except for age, which is presented as mean ± standard deviation. The *P* value for age was calculated using the Mann-Whitney U test. All other *P* values were derived from the χ² test or Fisher's exact test, as appropriate. EMVI, extramural venous invasion; MRF, mesorectal fascia invasion; NCRT, neoadjuvant chemoradiotherapy; TIL, tumor involvement of the intestinal lumen circumference; ypT0, pathological complete response, ypT1-4, pathological stage indicating residual tumor after neoadjuvant chemoradiotherapy.

### Diagnostic performance of CE MRI phases and interobserver agreement for restaging

**Figure 4:**
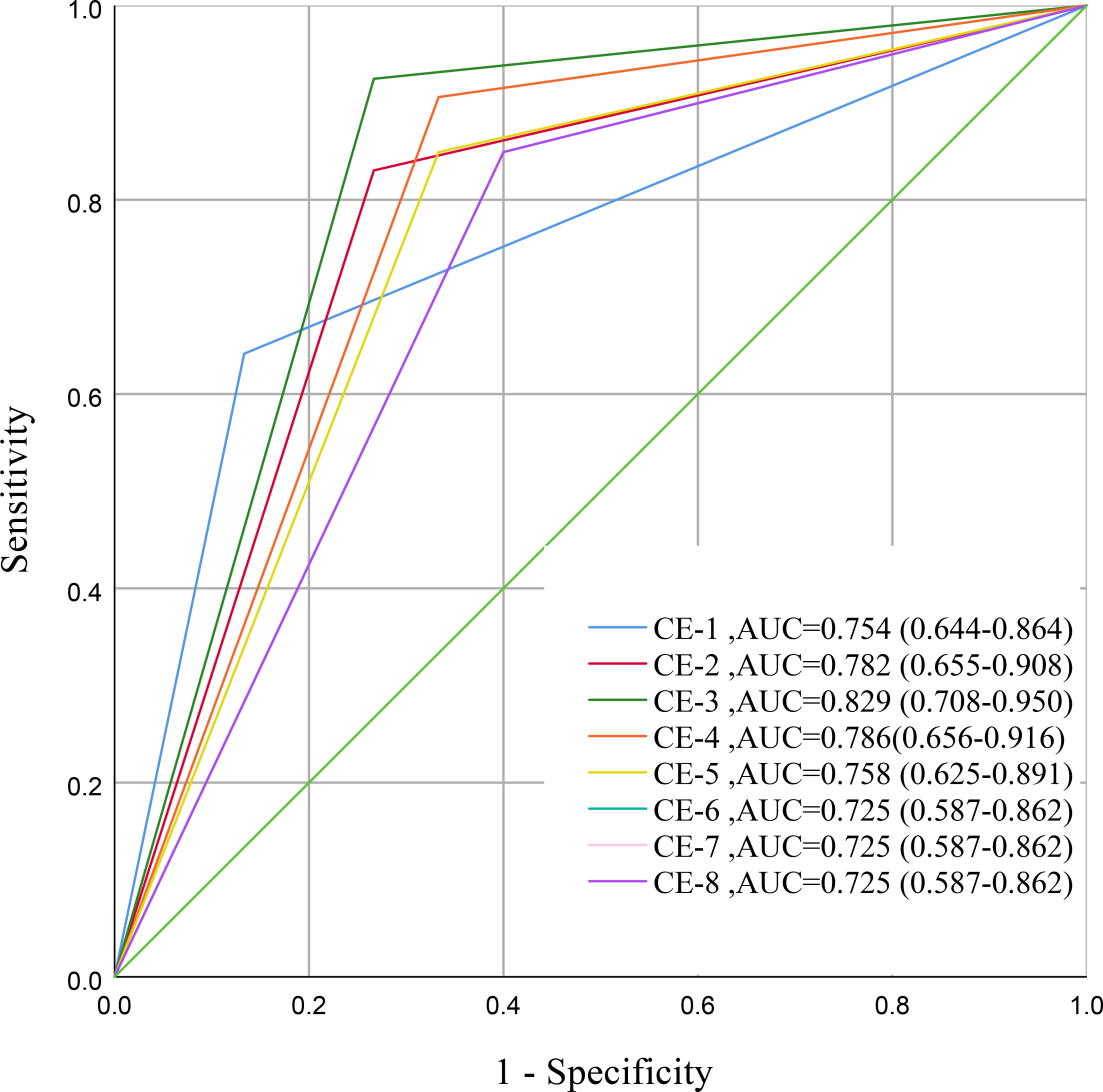
Comparison of diagnostic performance across phases of contrast-enhanced MRI. Receiver operating characteristic curves illustrate the performance of each contrast-enhanced phase in distinguishing ypT0 (pathological complete response) from ypT1-4 tumors.

The diagnostic performance of each of the eight dynamic CE phases for predicting ypT0 status is summarized in **Table 2**, **Figure 4**. Among all phases, the third phase (CE-3) demonstrated the most favorable and balanced diagnostic profile, achieving the highest accuracy (0.882, 60/68), specificity (0.925, 49/53), and area under the curve (AUC = 0.829; 95% CI: 0.708–0.950), with a sensitivity of 0.733 (11/15). Interobserver agreement for CE-3 was good (κ = 0.673; 95% CI: 0.461–0.886) (**Table 3**). Therefore, CE-3 was identified as the optimal phase for predicting pathologic complete response (representative multi-phase CE images are provided in **Supplementary Figure 1** and **2**).

**Table 2:** Diagnostic performance of multi-phase contrast-enhanced MRI (CE MRI) in predicting pathological complete response (ypT0).

Phase	Accuracy	Sensitivity	Specificity	PPV	NPV	AUC (95% CI)	*P* value
CE-1	0.691 (47/68)	0.867 (13/15)	0.642 (34/53)	0.406 (13/32)	0.944 (34/36)	0.754 (0.644-0.864)	<0.001
CE-2	0.809 (55/68)	0.733 (11/15)	0.830 (44/53)	0.550 (11/20)	0.917 (44/48)	0.782 (0.655-0.908)	<0.001
CE-3	0.882 (60/68)	0.733 (11/15)	0.925 (49/53)	0.733 (11/15)	0.925 (49/53)	0.829 (0.708-0.950)	<0.001
CE-4	0.853 (58/68)	0.667 (10/15)	0.906 (48/53)	0.667 (10/15)	0.906 (48/53)	0.786 (0.656-0.916)	<0.001
CE-5	0.809 (55/68)	0.667 (10/15)	0.849 (45/53)	0.556 (10/18)	0.900 (45/50)	0.758 (0.625-0.891)	<0.001
CE-6	0.794 (54/68)	0.600 (9/15)	0.849 (45/53)	0.529 (9/17)	0.882 (45/51)	0.725 (0.587-0.862)	0.001
CE-7	0.794 (54/68)	0.600 (9/15)	0.849 (45/53)	0.529 (9/17)	0.882 (45/51)	0.725 (0.587-0.862)	0.001
CE-8	0.794 (54/68)	0.600 (9/15)	0.849 (45/53)	0.529 (9/17)	0.882 (45/51)	0.725 (0.587-0.862)	0.001

Data are presented as the performance metric (number of correct predictions / total number of patients in the group), except for the area under the curve (AUC), which is shown as the point estimate with the 95% confidence interval (CI) in parentheses. CE, contrast-enhanced (phase number corresponds to the sequential order in the dynamic series); PPV, positive predictive value; NPV, negative predictive value. The *P* value corresponds to the statistical significance of the AUC being greater than 0.5 (null hypothesis).

**Table 3:** Interobserver agreement for T restaging across multi-phase contrast-Enhanced MRI (CE MRI).

Phase	Kappa (κ) statistic (95% CI)	*P* value
CE-1	0.509 (0.298-0.719)	<0.001
CE-2	0.471 (0.224-0.718)	<0.001
CE-3	0.673 (0.461-0.886)	<0.001
CE-4	0.625 (0.397-0.853)	<0.001
CE-5	0.530 (0.289-0.771)	<0.001
CE-6	0.530 (0.289-0.771)	<0.001
CE-7	0.480 (0.226-0.734)	<0.001
CE-8	0.423 (0.165-0.681)	<0.001

κ statistic (with 95% confidence interval, CI) was used to assess agreement between two independent readers for post-NCRT T staging on each phase of the dynamic contrast-enhanced MRI series. CE, contrast-enhanced (phase number corresponds to the sequential order in the dynamic series).

To further validate this selection, we compared CE-3 with each of the other seven phases using DeLong tests and NRI (**Supplementary Material Table 2**). No statistically significant difference was observed between CE-3 and CE-4 in terms of AUC (DeLong test, *P* = 0.217), overall NRI (p = 0.202), or NRI for the ypT0 group (*P* = 0.313). However, CE-3 showed significantly higher AUC than CE-6, CE-7, and CE-8 (DeLong test, *P* = 0.033 for each). Moreover, compared with CE-3, the NRI for the ypT1–4 (residual tumor) group was significantly negative for CE-1, CE-2, CE-5, CE-6, CE-7, and CE-8 (all *P* < 0.05), indicating that CE-3 correctly reclassified a larger proportion of patients with residual tumor.

In a separate analysis using CE-4 as the reference and excluding CE-3 (**Supplementary Material Table 3**), the only significant difference was found between CE-4 and CE-1 in the NRI for the ypT1–4 group (*P* < 0.001). No other significant differences were observed between CE-4 and the remaining phases (CE-2, CE-5, CE-6, CE-7, CE-8). These results indicate that while CE-4 performed similarly to CE-3 in direct comparisons, CE-3 demonstrated significant superiority over the majority of other phases (CE-1, CE-2, CE-5, CE-6, CE-7, CE-8), a pattern not observed for CE-4. This consistent and broader advantage supports the selection of CE-3 as the most robust phase.

Consequently, CE-3 was selected as the optimal CE phase for subsequent comparison with the T2DWI protocol.

### Comparison of the CE-3 protocol and the T2DWI protocol

**Figure 5:**
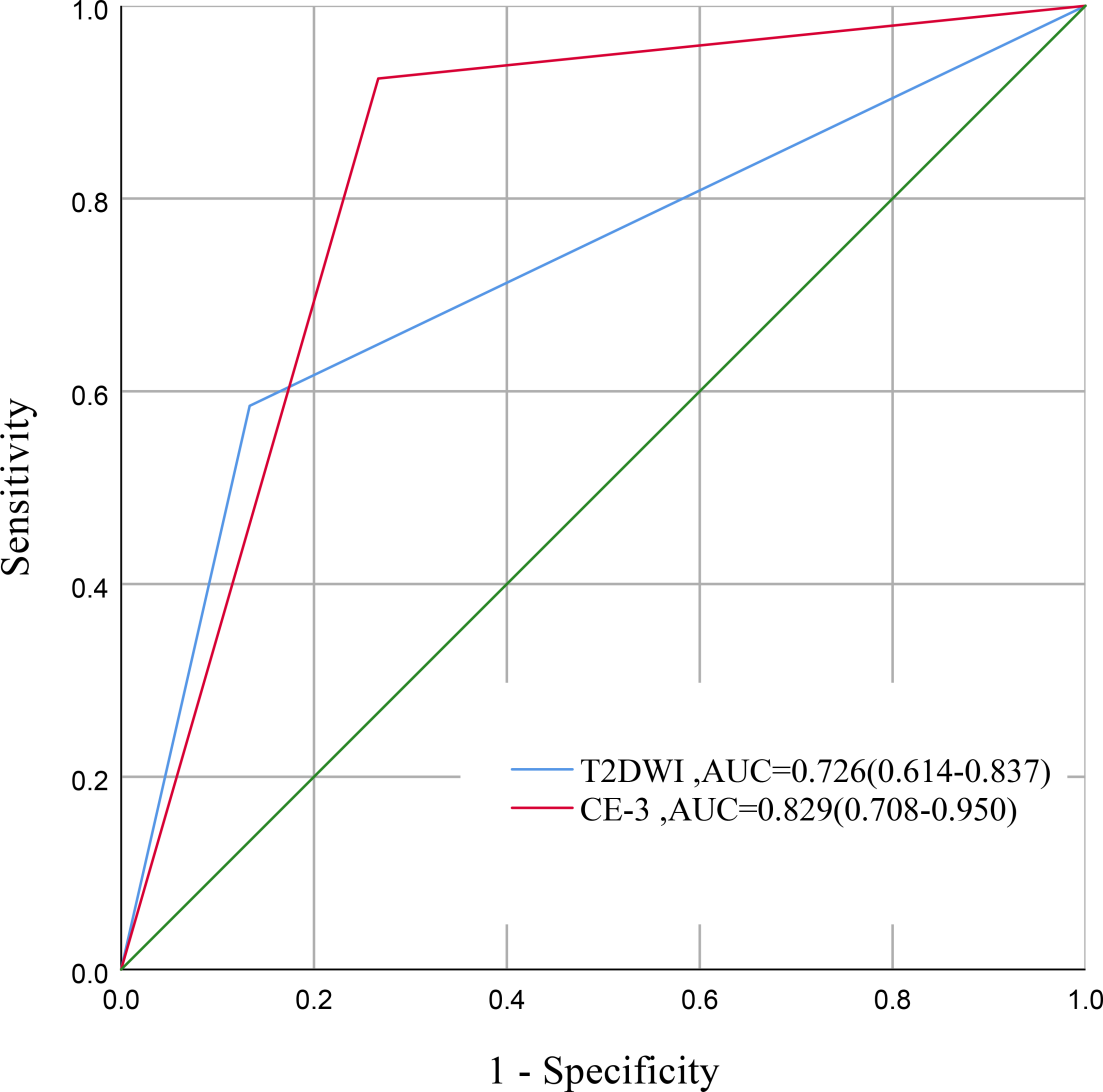
Comparison of diagnostic performance between CE-3 MRI and T2DWI. Receiver operating characteristic curves illustrate the performance of the third-phase contrast-enhanced MRI (CE-3) protocol and the conventional T2- weighted diffusion-weighted imaging (T2DWI) protocol in distinguishing ypT0 (pathologic complete response) from ypT1-4 tumors.

The overall diagnostic accuracy (0.882 vs. 0.647, *P* = 0.002) and specificity (0.925 vs. 0.585, *P* < 0.001) of the CE-3 protocol were significantly higher than those of the T2DWI protocol. The difference in sensitivity (0.733 vs. 0.867, *P* = 0.617) was not statistically significant. Comparison of AUC between CE-3 and T2DWI showed a higher value for CE-3 (0.829 vs. 0.726), though the DeLong test indicated no statistical significance (*P* = 0.172) (For details, see **Figure 5**; **Supplementary Table 4**). Further IDI and NRI tests revealed a statistically significant improvement in classification for the ypT1-4 (residual tumor) group using CE-3 (NRI = 0.339, *P* < 0.001) (**Table 4**). Good inter-observer agreement was observed for both protocols (CE-3 κ=0.673; T2DWI κ=0.726).

**Table 4:** Comparison of the CE-3 and T2DWI protocols for the prediction of pathological tumor stage.

Parameter	Estimate (95% CI)	*P* value
DeLong test (ΔAUC)	1.366(0.103)[-0.045–0.251]	0.172
Integrated Discrimination Improvement (IDI)	0.206(-0.111–0.514)	0.172
Net Reclassification Improvement (NRI)	0.206 (-0.095–0.490)	0.172
— NRI (ypT0 group)	-0.133 (-0.417–0.125)	0.506
— NRI (ypT1–4 group)	0.339(0.204–0.482)	<0.001

Data are presented as the point estimate with the 95% confidence interval (CI) in parentheses. For the DeLong test, the value in parentheses represents the standard error, followed by the 95% CI for the difference in the area under the curve (ΔAUC) between protocols. CE-3, the third phase of the contrast-enhanced MRI series; T2DWI, combined T2-weighted and diffusion-weighted MRI.

### Multivariable logistic regression analysis

Univariate logistic regression analysis was performed on all potential clinical and imaging variables (**Supplementary Table 5**). In the univariate analysis, only the post-NCRT yT stage assessed by the CE-3 protocol demonstrated a significant association with pCR (*P* < 0.001). No other variables, including pre-NCRT T stage, MRF, EMVI, or T2DWI assessment, met the prespecified entry criterion of *P* < 0.05.

Consequently, this single variable was entered into the multivariate logistic regression model. The analysis confirmed that yT staging based on CE-3 MRI was the sole independent predictor of pCR after NCRT, with an odds ratio of 22.782 (95% CI: 4.668–111.181; *P* < 0.001).

## Discussion

The “watch-and-wait” strategy may enable patients achieving pCR after NCRT for rectal cancer to avoid surgery and its associated complications (8, 9). Precise identification of patients attaining pCR is therefore paramount. This study systematically evaluated the predictive value of different time points in multi-phase CE MRI for pCR. The results demonstrated that CE-3 (approximately 76 seconds post-contrast) exhibited the most favorable diagnostic performance and reproducibility in predicting pCR, with an overall accuracy of 0.882, specificity of 0.925, AUC of 0.829, and κ = 0.673.

Multi-phase CE MRI non-invasively assesses microvascular permeability and perfusion status by tracking the dynamic distribution of contrast agents between vessels and tissues, based on signal intensity changes over time. Tumors promote endothelial cell proliferation and neovascularisation through secreted vascular endothelial growth factor (VEGF), exhibiting abnormal enhancement upon contrast agent injection, which indirectly reflects tumour generation and invasiveness (23, 24). The time-signal intensity curve comprises two phases: the wash-in phase, where the initial slope reflects local perfusion rate and angiogenesis; and the wash-out phase, whose morphology correlates with vascular permeability and interstitial pressure (25, 26). In assessing response to NCRT for rectal cancer, semi-quantitative analysis of CE-MRI has demonstrated potential predictive value, with favorable responders predominantly exhibiting descending wash-out curves (27, 28).

The superior diagnostic performance and inter-observer reproducibility of the third phase of contrast-enhanced imaging observed in this study (approximately 76 seconds post-injection) may be attributed to the contrast agent reaching peak perfusion within the tumor microvasculature and beginning to diffuse into the interstitial space at this time point. This is likely because residual tumor tissue, characterized by relatively intact microvascular structures and increased permeability, exhibits marked enhancement. In contrast, post-treatment fibrotic tissue shows delayed enhancement, as its transfer constant (K^trans) is negatively correlated with fibrotic content (29). This differential enhancement pattern maximizes visual contrast between viable tumor and treatment-induced fibrosis, thereby optimizing diagnostic discrimination.

The variation in diagnostic performance and inter-observer agreement across contrast-enhanced phases further supports these findings. In the early phase (CE-1), incomplete contrast extravasation and significant inter-individual hemodynamic differences cause inconsistent peak timing and enhancement degree. Moreover, the coexistence of residual tumor, inflammation, edema, and fibrosis after neoadjuvant chemoradiotherapy amplifies enhancement variability. These factors lead to highly unstable tumor-to-background contrast on CE-1: some residual tumors show only linear or weak enhancement, while true pCR patients may exhibit false-positive enhancement due to inflammatory hyperemia, risking misclassification of residual disease as pCR. Hence, CE-1 cannot reliably guide a “watch-and-wait” strategy. In intermediate phases (CE-2 to CE-5), residual tumors show progressive enhancement, fibrotic tissue enhances more slowly, and tumor-to-fibrosis contrast first increases then decreases. CE-3 captures the “window period” when tumor enhancement peaks while fibrotic enhancement is minimal, yielding maximal contrast. CE-2 shows incomplete tumor enhancement; CE-4/5 show increasing fibrotic enhancement, reducing contrast. Accordingly, CE-3 achieves the highest specificity for ypT0 (0.925), best inter-observer agreement (κ=0.673), and highest AUC (0.829, 95% CI 0.708–0.950), reaching the optimal balance. In contrast, late phases (CE-6 to CE-8) show declining performance, as contrast accumulates in fibrotic interstitium, reducing tumor-to-fibrosis contrast and obscuring residual disease.

To our knowledge, this study represents the first systematic comparison of all phases from multi-phase CE-MRI for predicting pCR in rectal cancer after NCRT. While previous studies have demonstrated the value of contrast-enhanced MRI in assessing treatment response—particularly the significance of mucosal linear enhancement in the arterial phase (19, 20)—none have specifically investigated which dynamic phase provides optimal diagnostic performance. Our finding that the third phase yields the highest predictive accuracy, specificity, and inter-observer agreement is therefore a novel contribution to the literature.

T2WI combined with DWI serves as the primary sequence for evaluating primary tumor response. However, post-NCRT, fibrosis within the primary tumor region and proliferating connective tissue often obscure small areas of moderately signal-intensity tumor tissue. This, coupled with T2 shine-through effects and magnetic susceptibility artifacts, frequently complicates assessment using T2DWI (21, 30). Studies employing T2DWI to evaluate complete and incomplete responses after NCRT for rectal cancer have demonstrated relatively low accuracy (e.g., 64%), with significant inter-reader variability in identifying complete responses (κ=0.24) (17), Inter-reader agreement for complete response after NCRT in rectal cancer has been reported as moderate (T2WI κ=0.56; DWI κ=0.54) (18). This study similarly observed that T2DWI demonstrated high sensitivity for assessing complete response but exhibited low specificity and positive predictive value (0.585, 0.371), potentially overestimating the complete response rates. This may lead to missed diagnoses in patients with residual tumor, thereby limiting its utility in guiding “watch-and-wait” strategies. Although current guidelines do not recommend CE MRI as an essential sequence for rectal MRI (31, 32), studies by Lu et al. and Miao et al. indicate that contrast enhancement can accurately assess post-NCRT ypT0–1 status, and MLE on CE MRI demonstrates high sensitivity in identifying pCR (19, 20). Similarly, our study found that CE-3 MRI protocol demonstrated superior overall accuracy (0.882 vs. 0.647, *P* = 0.002) and specificity (0.925 vs. 0.585, *P* < 0.001) when evaluating pCR compared to the T2DWI protocol. CE-3 MRI demonstrated a superior ability to correctly classify patients with residual tumor (ypT1–4 group NRI = 0.339, *P* < 0.001). In the multivariate logistic regression model, post-NCRT yT staging assessed solely by the CE-3 MRI emerged as the sole independent predictor of pCR (*P* < 0.001). This demonstrates that this specific phase of CE MRI can more accurately distinguish pCR from non-pCR patients, thereby potentially sparing pCR patients from overtreatment while preventing necessary treatment delays in non-pCR patients due to missed diagnoses.

This study has several limitations. First, its retrospective, single-center design and modest sample size—particularly the limited number of pCR cases (n=15)—resulted in reduced statistical power. However, the primary clinical value of CE-3 lies not in marginal AUC improvements but in its superior specificity (0.925 vs. 0.585, *P* < 0.001) and ability to correctly classify residual tumor (NRI = 0.339, *P* < 0.001). For “watch-and-wait” selection, a highly specific test that reliably rules out residual tumor is arguably more critical for patient safety than a marginal increase in sensitivity.

Second, as the first systematic comparison of all CE phases for pCR prediction, our findings lack direct external validation. The optimal timing (≈76 seconds) may be specific to our contrast protocol (2.0 mL/s, 0.1 mmol/kg) and 3.0T scanner. While the multi-metric superiority of CE-3 across accuracy, specificity, and inter-observer agreement provides internal consistency, these results are hypothesis-generating and require confirmation in larger, multicenter studies with standardized protocols.

Third, because patients with pCR had no identifiable enhancing tumor focus, reliable ROI-based semi-quantitative or quantitative CE-MRI analysis was not feasible. Therefore, our study relied on visual assessment, which reflects routine clinical practice. Future studies with dedicated CE-MRI protocols are needed to address this issue.

## Conclusion

CE MRI can identify residual tumor more precisely. In particular, the third phase of the contrast-enhanced MRI series demonstrated superior diagnostic efficacy (with the highest specificity and AUC) and good inter-observer agreement, which aids in identifying minute residual tumors. These findings suggest that the CE-3 protocol is a promising imaging biomarker for preoperative assessment, supporting safer patient selection for organ-preservation strategies, though its role requires further validation in larger cohorts.

## Data Availability

The data analyzed in this study is subject to the following licenses/restrictions: The datasets generated and/or analyzed during the current study are not publicly available due to patient privacy protection and institutional ethics restrictions. Access to de-identified data may be granted upon reasonable request and with approval from the Ethics Committee of The First Hospital of Putian City [Approval No. 2026-002]. Requests to access these datasets should be directed to JL, jjlinjiajun@163.com.
