# Supplementary material for: The predictive value of multi-phase contrast-enhanced MRI for pathological complete response after neoadjuvant chemoradiotherapy in rectal cancer
Source: Front Oncol. 2026 May 20;16:1834468. doi: 10.3389/fonc.2026.1834468 (PMC13229763; doi:10.3389/fonc.2026.1834468)
Supplement: Supplementary file 1 [file DataSheet1.docx]

| **Supplementary Table 1: MRI Sequence Parameters for Rectal Imaging** | | | | |
| --- | --- | --- | --- | --- |
| Parameter | T2WI Dixon (large FOV) | High-Resolution Axial T2WI | Dynamic T1 VIBE Dixon | DWI |
| **Acquired voxel (mm³)** | 1.1 × 0.9 × 5.0 | 0.9 × 0.7 × 3.0 | 1.3 × 0.9 × 4.4 | 2.2 × 2.2 × 3.0 |
| **Reconstructed voxel (mm³)** | 0.9 × 0.9 × 5.0 | 0.7 × 0.7 × 3.0 | 0.9 × 0.9 × 3.0 | 1.1 × 1.1 × 3.0 |
| **Flip angle (°)** | 90 | 90 | 13 | 90 |
| **FOV (mm²)** | 404 × 340 | 220 × 220 | 280 × 280 | 260 × 205 |
| **Slice gap (mm)** | 1 | 0 | 0 | 0 |
| **Number of slices** | 30 | ≥30 | 44 | ≥30 |
| **Phase encoding direction** | RL | RL | RL | AP |
| **TR (ms)** | 3370 | 2690 | 5.63 | 6100 |
| **TE / TE1/TE2 (ms)** | 93 | 103 | 2.46 / 3.69 | - |
| **Scan time (s)** | 125 | 186 | 184 | 240 |
| **Generated images** | In-/opposed-phase, fat, water | - | Fat, water | ADC map, b=1500 s/mm² |
| **Note. —** DWI = diffusion-weighted imaging, FOV = field of view, TR = repetition time, TE = echo time, VIBE = volumetric interpolated breath-hold examination, RL= right–left, AP= anterior–posterior. | | | | |

| **Supplementary Table 2: Comparison of Diagnostic Performance and Reclassification Metrics Between CE‑3 and Other Contrast‑Enhanced Phases for Predicting Pathologic Complete Response (ypT0)** | | | | | |
| --- | --- | --- | --- | --- | --- |
| Phase | AUC (95% CI) | DeLong *P* | Overall NRI (*P*) | NRI for ypT0 (*P*) | NRI for ypT1-4 (*P*) |
| CE-3 | 0.829 (0.708–0.950) | - | - | - | - |
| CE-1 | 0.754 (0.644–0.864) | 0.309 | -0.150 (0.295) | 0.133(0.301) | -0.283 (<0.001) |
| CE-2 | 0.782 (0.655–0.908) | 0.372 | -0.094 (0.357) | 0.000(1.000) | -0.094 (0.019) |
| CE-4 | 0.786 (0.656–0.916) | 0.217 | -0.086 (0.202) | -0.067(0.301) | -0.019 (0.313) |
| CE-5 | 0.758 (0.625–0.891) | 0.062 | -0.142 (0.055) | -0.067(0.301) | -0.075 (0.038) |
| CE-6 | 0.725 (0.587–0.862) | 0.033 | -0.209 (0.028) | -0.133(0.129) | -0.075 (0.038) |
| CE-7 | 0.725 (0.587–0.862) | 0.033 | -0.209 (0.028) | -0.133(0.129) | -0.075 (0.038) |
| CE-8 | 0.725 (0.587–0.862) | 0.033 | -0.209 (0.028) | -0.133(0.129) | -0.075 (0.038) |
| **Note.** – DeLong test and Net Reclassification Improvement (NRI) are shown for CE‑3 versus each other phase. Overall NRI, NRI for the ypT0 group , and NRI for the ypT1–4 group are presented with associated P values in parentheses. Bold indicates statistical significance (P < 0.05). AUC = area under the receiver operating characteristic curve, CI = confidence interval, CE = contrast‑enhanced (phase number indicates the order in the dynamic series). | | | | | |

| Supplementary Table 3: Comparison of Diagnostic Performance and Reclassification Metrics Between CE‑4 and Other Contrast‑Enhanced Phases (Excluding CE‑3) for Predicting Pathologic Complete Response (ypT0) | | | | | |
| --- | --- | --- | --- | --- | --- |
| Phase | AUC (95% CI) | DeLong *P* | Overall NRI (*P*) | NRI for ypT0 (*P*) | NRI for ypT1-4 (*P*) |
| CE-4 | 0.786 (0.656–0.916) | - | - | - | - |
| CE-1 | 0.754 (0.644–0.864) | 0.683 | -0.064 (0.674) | 0.200 (0.153) | -0.264 (<0.001) |
| CE-2 | 0.782 (0.655–0.908) | 0.945 | -0.009 (0.943) | 0.067 (0.559) | -0.075 (0.094) |
| CE-5 | 0.758 (0.625–0.891) | 0.077 | -0.057 (0.075) | 0.000 (NA) | -0.057 (0.075) |
| CE-6 | 0.725 (0.587–0.862) | 0.096 | -0.123 (0.086) | -0.067 (0.301) | -0.057 (0.075) |
| CE-7 | 0.725 (0.587–0.862) | 0.096 | -0.123 (0.086) | -0.067 (0.301) | -0.057 (0.075) |
| CE-8 | 0.725 (0.587–0.862) | 0.096 | -0.123 (0.086) | -0.067 (0.301) | -0.057 (0.075) |
| **Note.** – CE‑3 is excluded from this comparison (its performance is shown in Table 1). CE‑4 was used as the reference phase. DeLong test and Net Reclassification Improvement (NRI) are shown for CE‑4 versus each other phase. Overall NRI, NRI for the ypT0 group, and NRI for the ypT1–4 group are presented with associated P values in parentheses. NA = not applicable (zero reclassification in one category). Bold indicates statistical significance (P < 0.05). AUC = area under the receiver operating characteristic curve, CI = confidence interval, CE = contrast‑enhanced (phase number indicates the order in the dynamic series). | | | | | |

| **Supplementary Table 4: Diagnostic Performance of CE-3 and T2DWI in Predicting ypT0 Tumors** | | | | | | | |
| --- | --- | --- | --- | --- | --- | --- | --- |
| Parameter | Accuracy | Sensitivity | Specificity | PPV | NPV | AUC | *P* Value |
| CE-3 | 0.882（60/68） | 0.733 (11/15) | 0.925 (49/53) | 0.733 (11/15) | 0.925 (49/53) | 0.829 [0.708-0.950] | ＜0.001 |
| T2DWI | 0.647（44/68） | 0.867 (13/15) | 0.585 (31/53) | 0.371 (13/35) | 0.939 (31/33) | 0.726 [0.614-0.837] | ＜0.001 |
| **Note.—** Except where indicated, data in parentheses are numerators/denominators. Data in brackets are 95% CIs. AUC = area under the receiver operating characteristic curve, NPV = negative predictive value, PPV = positive predictive value. CE-3 = the third phase of contrast enhanced MRI, T2DWI= T2-weighted imaging combined with diffusion-weighted MRI | | | | | | | |

| **Supplementary Table 5: Univariable Logistic Regression Analysis of Factors Associated with ypT0 Status** | | | | | |
| --- | --- | --- | --- | --- | --- |
| Variable | Reference / Comparator | Odds Ratio | 95% CI Lower | 95% CI Upper | *P* Value |
| **Sex** |  |  |  |  |  |
| Female | Male | 3.341 | 0.994 | 11.230 | 0.051 |
| **Age (per 1-year increase)** |  | 0.986 | 0.922 | 1.53 | 0.670 |
| **Tumor location (vs. Low)** |  |  |  |  |  |
| Middle | Low | 0.853 | 0.239 | 3.043 | 0.807 |
| Upper | Low | 0.914 | 0.142 | 5.902 | 0.925 |
| **Pre-NCRT T stage (vs. T2)** |  |  |  |  |  |
| T3 | T2 | 0.606 | 0.128 | 2.873 | 0.528 |
| T4 | T2 | 0.286 | 0.038 | 2.173 | 0.226 |
| **Tumor involvement (vs. <1/2)** |  |  |  |  |  |
| 1/2–3/4 | <1/2 | 0.571 | 0.075 | 4.346 | 0.589 |
| >3/4 | <1/2 | 0.514 | 0.081 | 3.267 | 0.481 |
| **Pre-NCRT MRF status** |  |  |  |  |  |
| Negative | Positive | 0.939 | 0.292 | 3.023 | 0.917 |
| **Pre-NCRT EMVI status** |  |  |  |  |  |
| Negative | Positive | 0.578 | 0.143 | 2.332 | 0.441 |
| **Post-NCRT MRF status** |  |  |  |  |  |
| Negative | Positive | 0.402 | 0.046 | 3.497 | 0.409 |
| **Post-NCRT EMVI status*** |  |  |  |  |  |
| Negative | Positive | 0.000 | 0.000 | - | 0.991 |
| Pre-NCRT MRI N stage |  |  |  |  |  |
| N0 | N1 | 0.604 | 0.182 | 2.010 | 0.411 |
| Post-NCRT MRI N stage* |  |  |  |  |  |
| N0 | N1 | 0.000 | 0.000 | - | 0.993 |
| **MRI assessment** |  |  |  |  |  |
| T2DWI (yT0 vs. yT1-4) | yT1-4 | 9.159 | 1.876 | 44.726 | 0.006 |
| CE-3 (yT0 vs. yT1-4) | yT1-4 | 33.687 | 1.687 | 155.958 | **<0.001** |
| **Note.—**CI = confidence interval, CE-3 = the third phase of contrast enhanced MRI, EMVI = extramural venous invasion, MRF = mesorectal fascia invasion, NCRT = neoadjuvant chemoradiotherapy, T2DWI = T2-weighted imaging combined with diffusion-weighted imaging. *The odds ratio could not be calculated for this variable due to zero cells in the contingency table (no patients with ypT0 had post-NCRT EMVI) | | | | | |


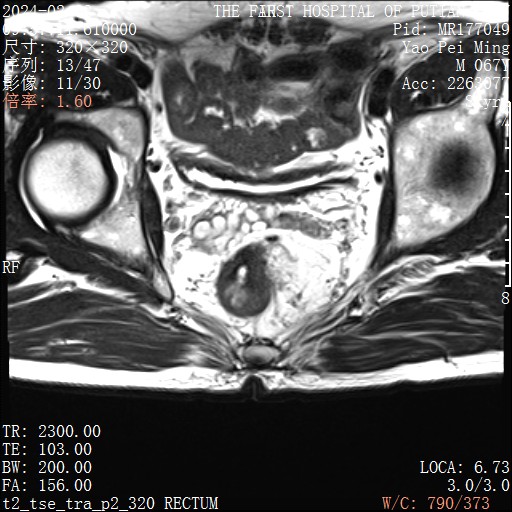

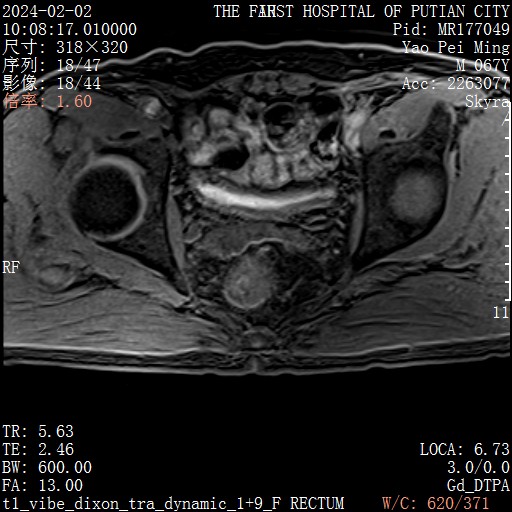

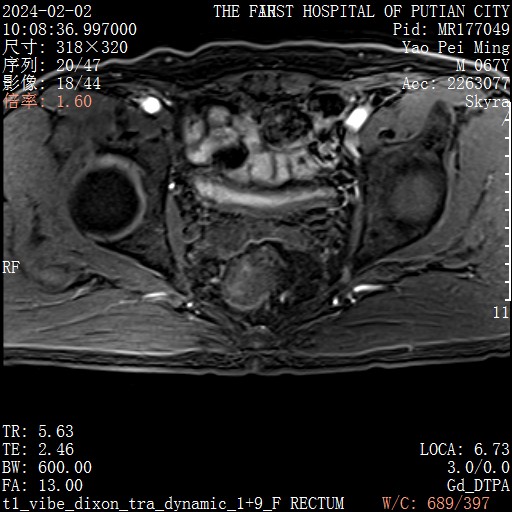

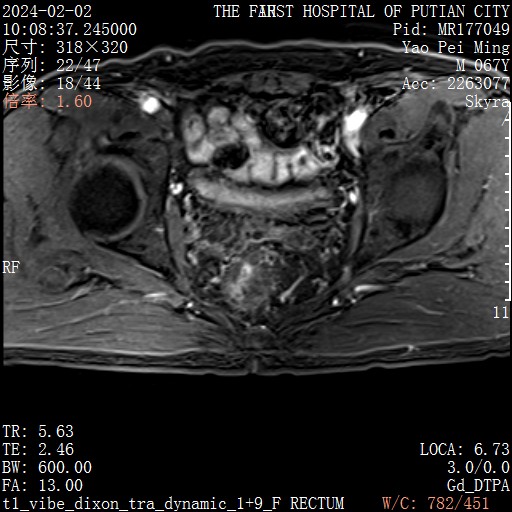

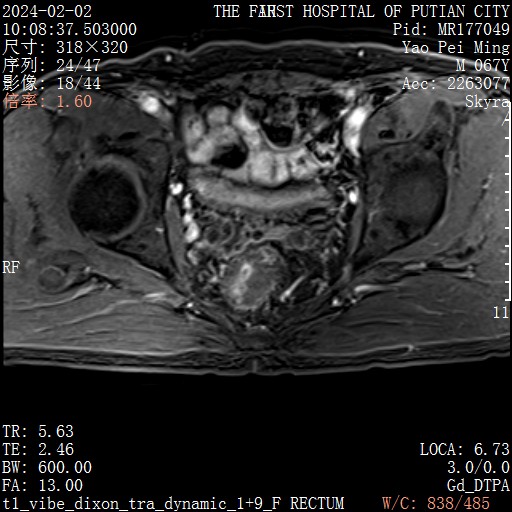

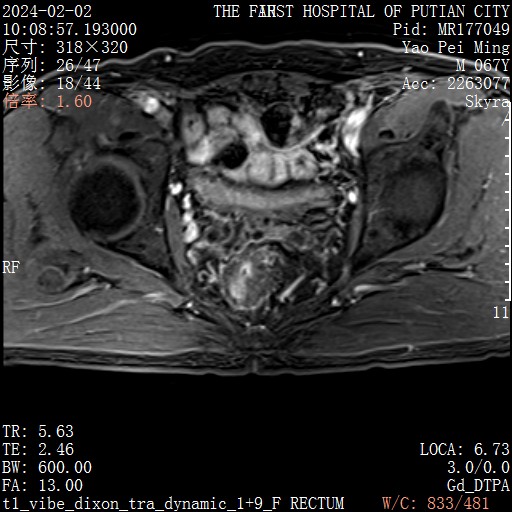

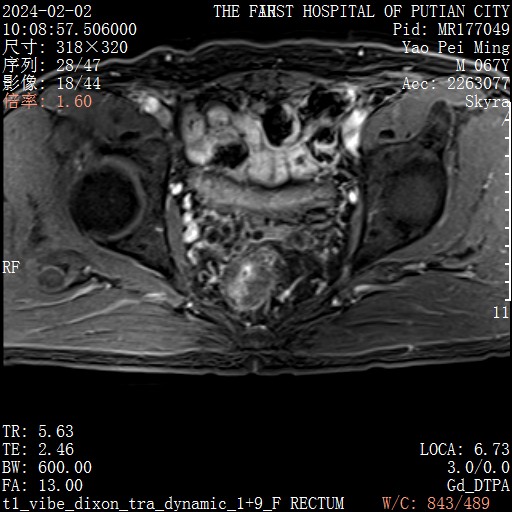

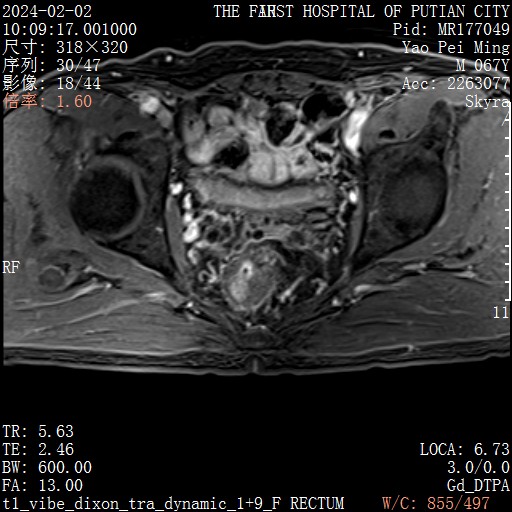

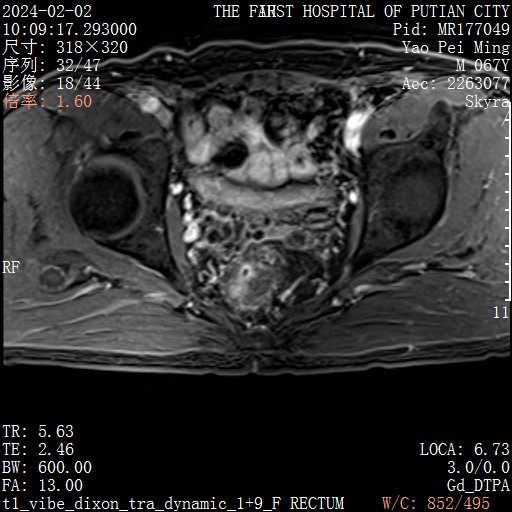

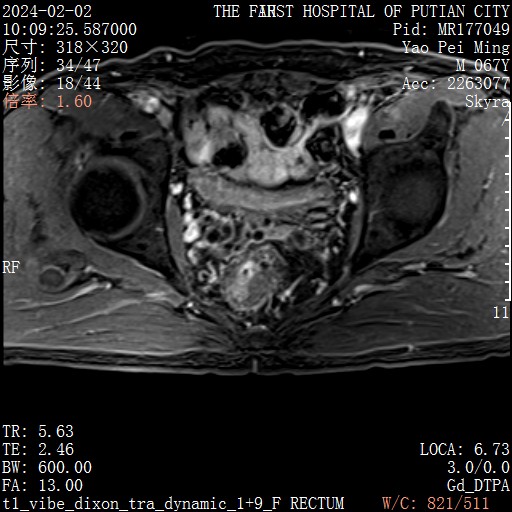

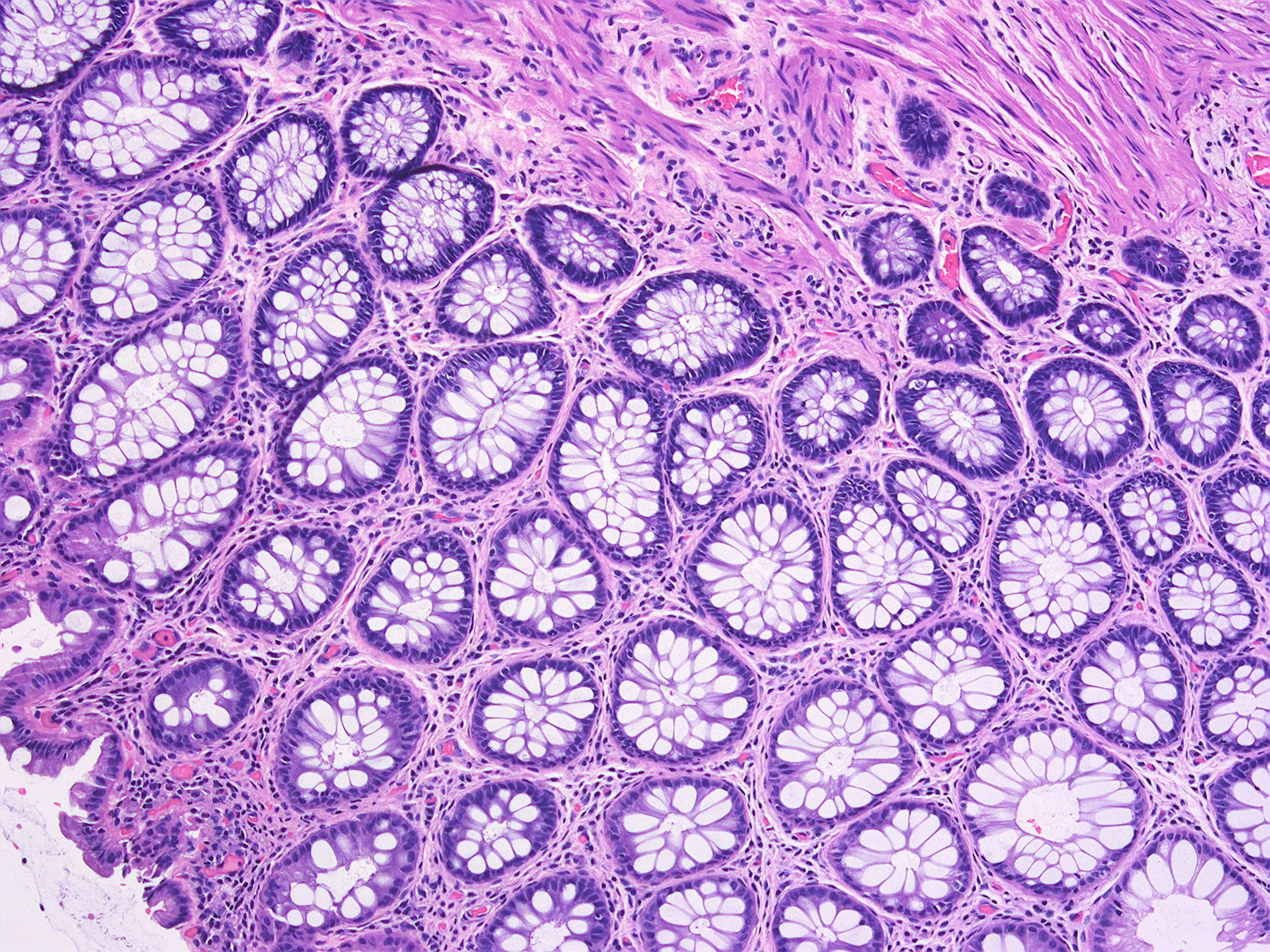


**A**

**B**

**C**

**D**

**E**

**F**

**G**

**H**

**I**

**J**

**K**

**Supplementary Figure 1:** A 67-year-old male patient with mid-rectal cancer who achieved pathologic complete response (pCR) after neoadjuvant chemoradiotherapy.(A) Unenhanced axial T1-weighted fat-suppressed image.(B–I) Axial fat-suppressed T1-weighted images from eight dynamic contrast-enhanced phases (CE-1 to CE-8).(D) CE-3 (approximately 76 seconds after contrast injection) shows thin linear mucosal enhancement within the original tumor bed indicating complete response.(B, C) The early phases (CE-1 and CE-2) show more extensive and conspicuous enhancement due to inflow effects and delayed contrast arrival, which may overestimate residual tumor.(E–I) The intermediate to late phases (CE-4 to CE-8) demonstrate progressive enhancement of post-treatment fibrosis, leading to a larger area of enhancement that could be misinterpreted as residual disease.(J) Axial T2-weighted image after neoadjuvant therapy shows no definite tumor signal.(K) Postoperative pathological examination of the mesorectal specimen confirmed a complete pathological response, with no residual tumor tissue identified.


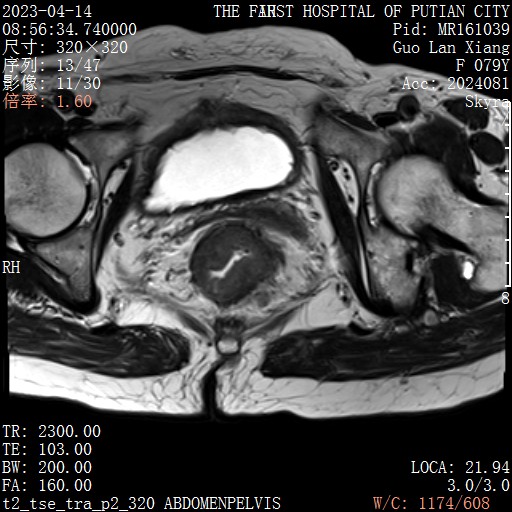

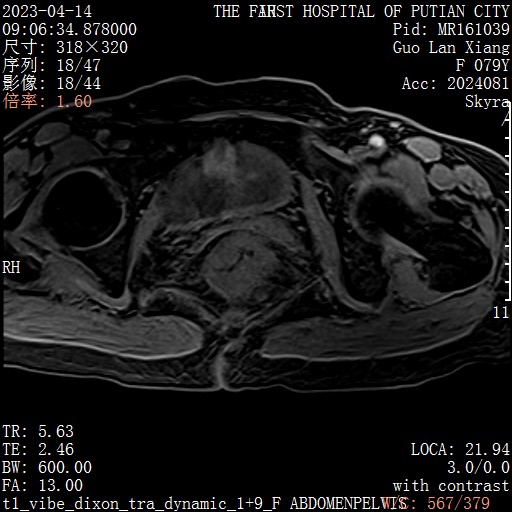

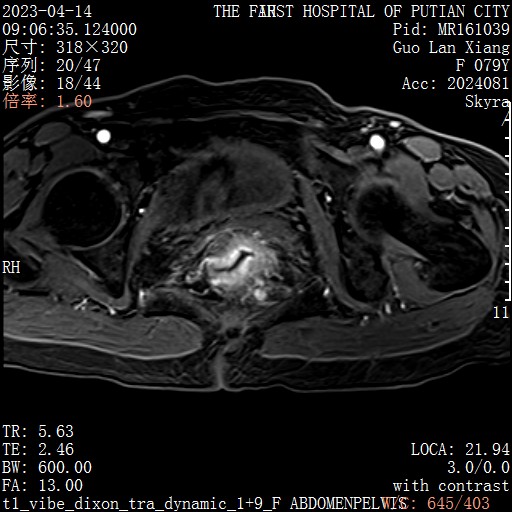

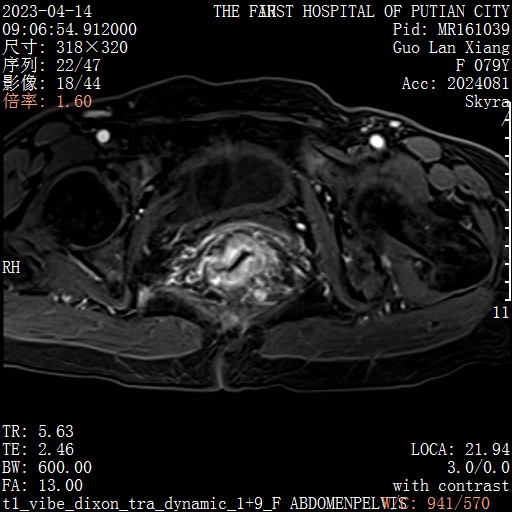

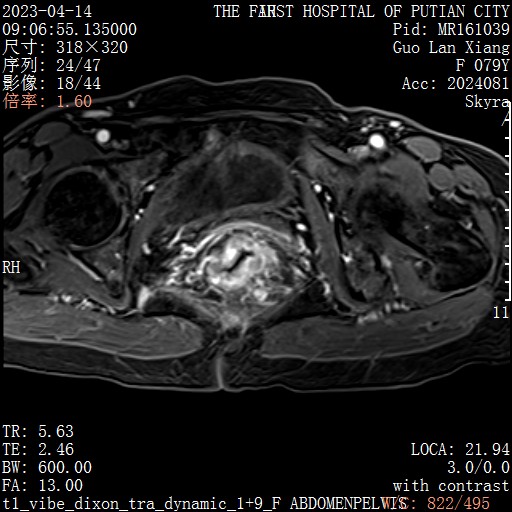

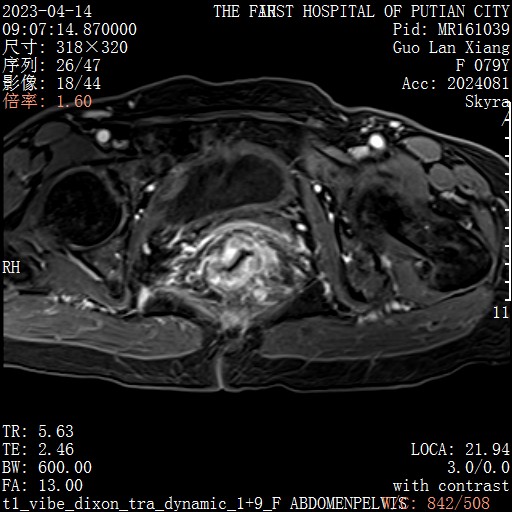

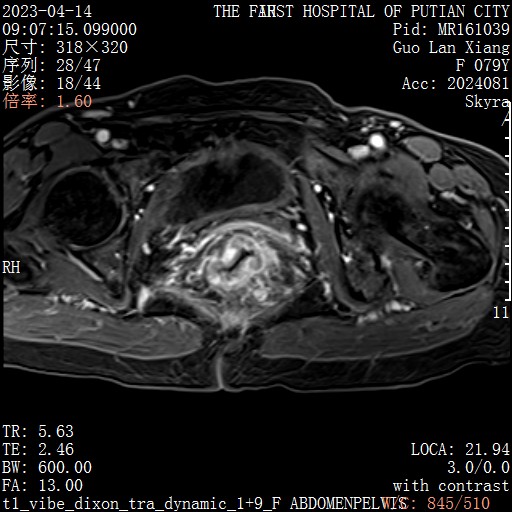

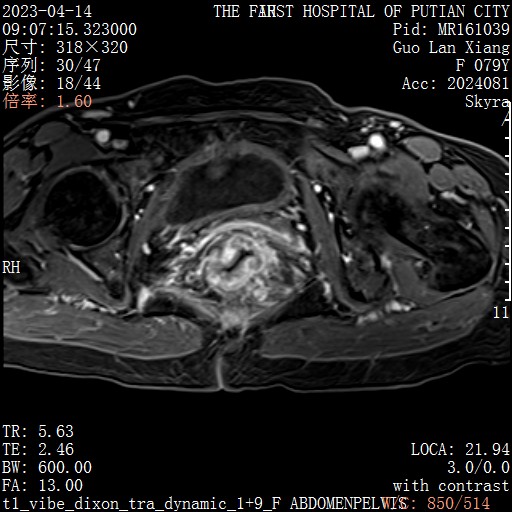

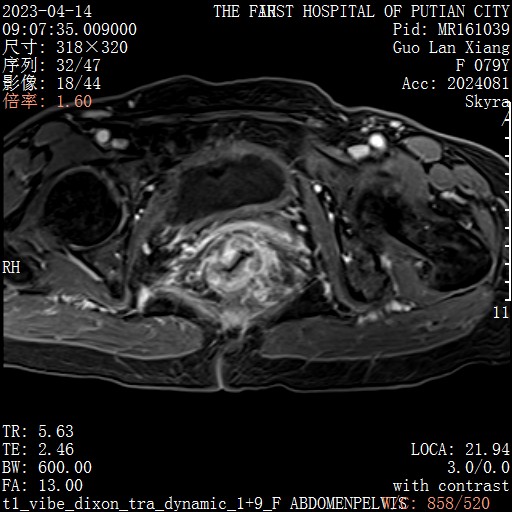

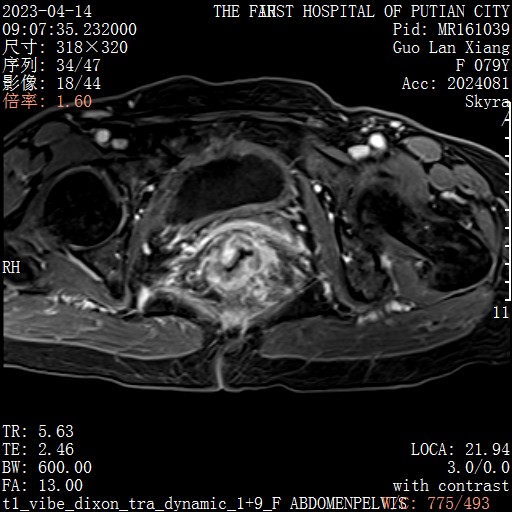

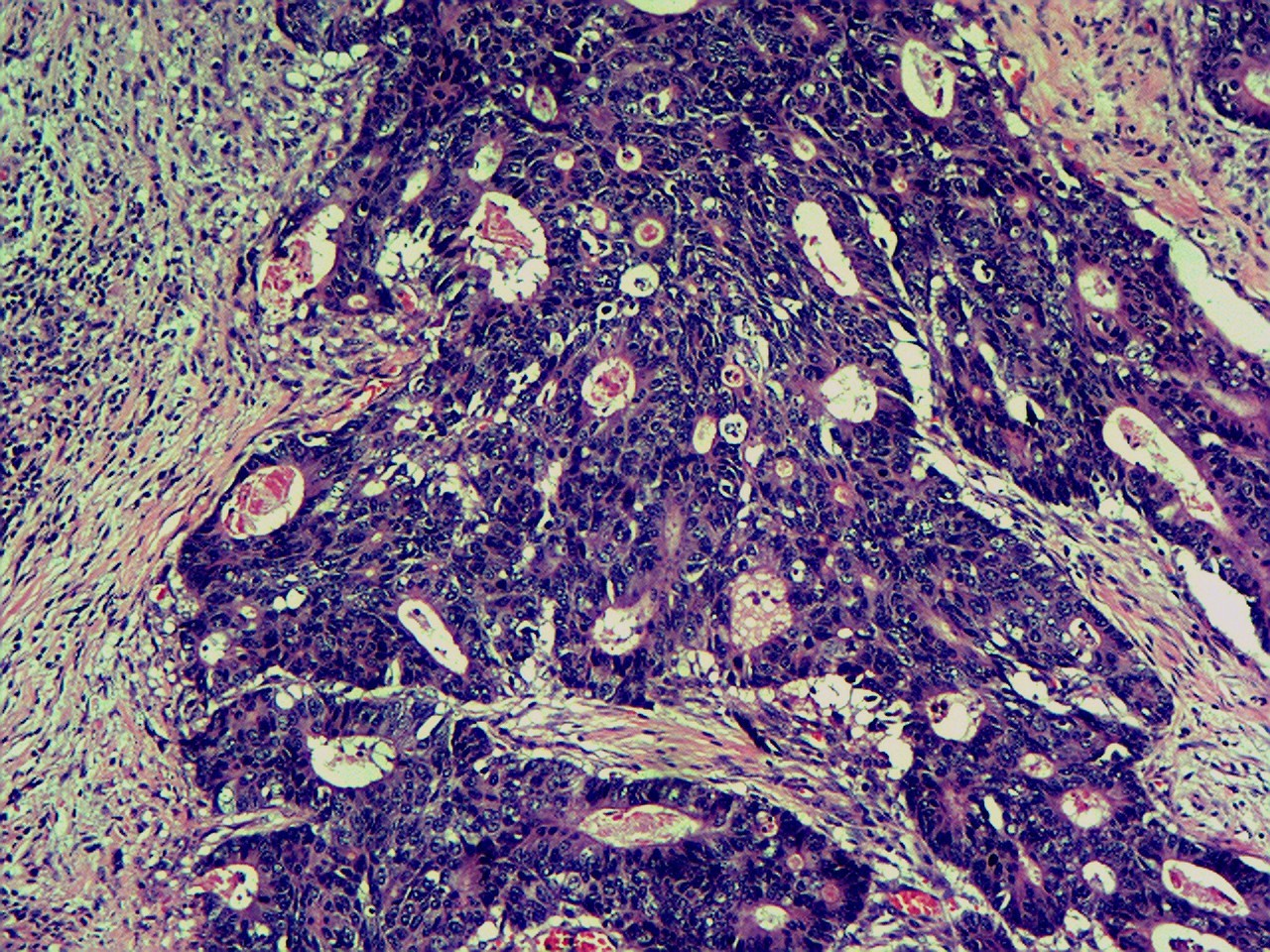


**A**

**E**

**I**

**B**

**F**

**J**

**C**

**G**

**K**

**D**

**H**

**Supplementary Figure 2:** A 79-year-old female patient with low rectal cancer who had residual tumor after neoadjuvant chemoradiotherapy.(A) Unenhanced axial T1-weighted fat-suppressed image.(B–I) Axial fat-suppressed T1-weighted images from multiple dynamic contrast-enhanced phases (CE-1 to CE-8).(D) CE-3 (approximately 76 seconds after contrast injection) clearly delineates the extent of the residual tumor with sharp margins.(B, C) The early phases (CE-1 and CE-2) show incomplete tumor delineation due to insufficient contrast filling.(E–I) The intermediate to late phases (CE-4 to CE-8) demonstrate progressive enhancement of post-treatment fibrosis, leading to obscured tumor margins and an apparent increase in the enhanced area, which may overestimate the extent of residual disease.(J) Axial T2-weighted image after neoadjuvant therapy shows obvious residual tumor invading the perirectal fat, with a lymph node visible posterior to the left side of the lesion.(K) Photomicrograph confirms the presence of abundant residual tumor cells.
